# Executive function profiles of preschool children with autism spectrum disorder and attention‐deficit/hyperactivity disorder: A systematic review

**DOI:** 10.1002/jcv2.12123

**Published:** 2023-01-07

**Authors:** Marina Christoforou, Emily J. H. Jones, Philippa White, Tony Charman

**Affiliations:** ^1^ Department of Psychology Institute of Psychiatry, Psychology and Neuroscience King's College London London UK; ^2^ Centre for Brain and Cognitive Development Birkbeck University of London London UK; ^3^ Department of Child Psychiatry Institute of Psychiatry, Psychology and Neuroscience King's College London London UK

**Keywords:** attention deficit hyperactivity disorder, autism, executive function, preschool

## Abstract

**Background:**

Autism Spectrum Disorder (ASD) and Attention‐Deficit/Hyperactivity Disorder (ADHD) are both associated with differences in Executive Functioning (EF). There is lack of clarity around the specificity or overlap of EF differences in early childhood when both disorders are first emerging.

**Method:**

This systematic review aims to delineate preschool EF profiles by examining studies comparing the EF profiles of children with and without ASD or ADHD. Five electronic databases were systematically searched (last search in May 2022) to identify published, quantitative studies of global and specific EF (Inhibition, Shifting, Working Memory (WM), Planning and Attentional Control), comparing children aged 2‐6 with a diagnosis of ASD or ADHD to peers without ASD or ADHD.

**Results:**

Thirty‐one empirical studies (10 ADHD and 21 ASD studies) met criteria for inclusion. EF profiles in preschool ASD were characterised by consistent Shifting, and, in most cases, Inhibition impairments. ADHD studies consistently reported impairments in Inhibition and Planning, and in most cases WM. Findings with regards to sustained Attention and Shifting in ADHD and WM and Planning in ASD were mixed.

**Conclusions:**

Overall, current evidence indicates overlap but also some specificity in EF impairments in preschool ASD and ADHD. There were differences in the degree to which individual domains were impaired, with Shifting more consistently impaired in ASD, and Inhibition, WM and Planning in ADHD. Methodological issues and differences in methods of outcome measurement could potentially underlie mixed findings, as informant‐based measures revealed more robust EF impairments than laboratory‐based tasks.

**Key points**

- Both Autism Spectrum Disorder (ASD) and ADHD have been associated with impairments in Executive Functioning (EF), but the profiles of those impairments during early childhood are not well established.
- Preschoolers with ASD and preschoolers with ADHD over 4 years of age demonstrate robust impairments in global EF.
- Preschoolers with ASD appear to be consistently impaired mainly in Shifting, while preschoolers with ADHD are more consistently impaired in Inhibition, Planning and Working Memory.
- Early interventions addressing those domains in young children with ASD and ADHD might be beneficial in preventing further impairments in executive skills which are crucial for everyday functioning, attainment, as well as mental and emotional wellbeing.

## INTRODUCTION

### Executive functioning in ASD and ADHD

Autism Spectrum Disorder/ASD and Attention Deficit Hyperactivity Disorder/ADHD are neurodevelopmental conditions that typically emerge in childhood (Rutter et al., 2006), are highly heritable and often co‐occur (Rommelse et al., 2010a; Simonoff et al., 2008). Delineating the early neuropsychological profiles of ASD and ADHD may help elucidate shared and distinct processes that underlie observable neurodevelopmental features and associated difficulties (Johnson et al., 2015) and provide targets for early intervention (Constantino et al., 2021). One important neuropsychological domain that has been implicated in etiological models of both ASD and ADHD, is executive functioning (EF), as a shared endophenotype (Rommelse et al., 2011) or protective factor (Johnson, 2012). EF is essential for social, occupational and academic functioning, physical and mental health and quality of life, and comprises top‐down neuropsychological functions such as inhibition (of behaviour, attention or cognition in order to achieve a goal), shifting (changing internal perspectives or adjusting behaviour to new demands), and working memory, that is, mentally manipulating information held in mind (Diamond, 2013). Built on these domains, higher‐order executive processes (problem‐solving and planning) underpin decision‐making and behaviour (Collins & Koechlin, 2012). A meta‐analysis by Demetriou et al. (2018) confirmed that, compared to neurotypical controls, children and adults with ASD are more likely, as a group, to exhibit a broad EF impairment, which was found to be relatively stable across development. Further, both ASD and ADHD have been associated with abnormalities in the prefrontal cortex, which is linked to EF (Friedman & Robbins, 2022). Mapping EF skills may be important in understanding the developmental paths shaping the co‐occurrence of ASD and ADHD in early development.

### The importance of the preschool period

Most work on EF in ASD and ADHD has focused on either a broad age range (e.g. Demetriou et al., 2018) or middle childhood and adolescence (Craig et al., 2016; Geurts et al., 2014; Willcut et al., 2005). Studies show some differentiation between how specific domains of EF are affected in the two conditions; for example, in a review covering 3‐18 year‐olds, shifting and planning deficits were more common in ASD, whilst inhibition deficits were more apparent in ADHD (Craig et al., 2016). However, there is also substantial heterogeneity in EF profiles within older children with ASD or ADHD (e.g. Geurts et al., 2014), indicating that it is unlikely that EF would yield specific diagnostic markers for either condition. More recently, there has been increased interest in the study of EF during the preschool years (which for the purposes of the present study we define at ≤ 6 years of age). This is because the executive system and its associated brain structures undergo significant changes during early childhood (Johnson et al., 2015). It has also been proposed that the foundation of EF skills is set during the preschool period (Garon et al., 2008), with individual differences in attention control and behavioural inhibition starting to become more stable around the end of the first year, and individual differences in shifting becoming more stable after 24 months (see Hendry et al., 2016; for a review). EF skills in early childhood can predict later socio‐emotional adjustment and school readiness (Best et al., 2011), may be sensitive to changes in the environment and improve with training (Scionti et al., 2020). Although there is debate about whether EF is better conceptualised as a unitary construct at this stage in development (e.g. Howard et al., 2015), there is some evidence to suggest there are separate EFs at this age (e.g. Miller et al., 2012).

The preschool period is also important with regards to identifying the first signs of ASD and ADHD. ASD can be reliably diagnosed in some cases from about the second year of life (Ozonoff et al., 2015; Yirmiya & Charman, 2010), while ADHD behaviours become predictive of later ADHD psychopathology slightly later in the preschool period (Leblanc et al., 2008). Both ASD and ADHD are neurodevelopmental conditions associated with a range of etiological factors that are present prenatally, and prospective studies of infants with a family history of ASD and ADHD show that early behavioural changes are apparent from around 12 months of age (Jones et al., 2014; Miller et al., 2021; Szatmari et al., 2016; Tobarra‐Sanchez et al., 2022). However, by studying EF profiles in the preschool period when the full clinical profiles of ASD and ADHD are still emerging, EF differences may be less affected by compensatory or cascading effects of clinically diagnostic symptoms and thus any specificity of profile to either condition may be more clearly seen than in school age children.

### The current review

One previous narrative review (Visser et al., 2016) compared EF outcomes in ASD with those in ADHD in subclinical and clinical samples of infants and preschoolers. They found that impairments in shifting are particularly prominent and appear first in ASD, while impairments in inhibition are detected earlier and have stronger associations with ADHD. Similar to reviews of older children, they noted mixed findings, which they attributed to discrepancies in participant age and outcome measurement. To provide an updated picture, the aim of the current systematic review is to delineate the EF profiles of preschool children with a clinical diagnosis of either ADHD or ASD compared to children with typical development or other conditions, and note similarities and differences. The demographics, matching criteria and methods of measuring EF will be taken into account when synthesising the findings, given the inconsistencies identified in previous reviews.

## METHODS

### Protocol and registration

The current systematic review was added to the PROSPERO register on 30/06/2020 (CRD42020189409). The review was carried out in line with the PRISMA guidelines (Page et al., 2021).

### Eligibility criteria

Records were included if they met the following criteria: (1) Published, peer‐reviewed studies written in English; (2) Empirical studies comparing two or more groups of participants; (3) Studies include a clinical group with a diagnosis of either ASD or ADHD. The diagnosis must have been made using a standardised diagnostic instrument or based on established diagnostic criteria (DSM or ICD; American Psychiatric Association, 2013; World Health Organization, 1993); (4) The mean age for the whole sample is between 2 and 6 years, in order to cover different countries' definition of the preschool period/early childhood; (5) If the study compares multiple clinical groups, the results for participants with ASD and/or ADHD must be reported separately; (6) The comparison group comprises preschool children without a diagnosis of ASD or ADHD. Studies directly comparing outcomes of children with ASD to those of children with ADHD should include an additional comparison group (without ASD/ADHD); (7) Studies have a minimum sample size of 15 per group.^1^; (8) The study measures “cool” EF domains, that is, those measured in affectively neutral tasks (see Zelazo & Carlson, 2012), such as cognitive/behavioural Inhibition, Shifting, WM, Planning/Problem‐solving, Attentional control, or Global EF.

### Exclusion criteria

(1) Grey literature, book chapters, commentaries, letters or conference abstracts; (2) Qualitative studies; (3) Studies published in a different language/not available in English. (4) Longitudinal/prospective studies that do not include cross‐sectional comparisons between the different groups; (5) Studies measuring only “hot” EF (e.g. Emotion Control) or studies focusing on EF‐related temperament constructs (e.g. Effortful Control, Self‐Regulation). (6) Studies of participants without a diagnosis of ASD/ADHD (e.g. at‐risk or subclinical samples of children with symptoms of ASD/ADHD).

### Information sources and search strategy

Studies were primarily identified by searching electronic databases (PubMed, MEDLINE, EMBASE, Web of Science, PsycINFO). The reference lists of included articles were also checked. Where possible, searches were restricted to child populations and to studies published in peer‐reviewed journals in the English language (see Supplementary Materials Tables S1–S4 for search terms optimised for each database). Initial searches were run in May 2020 and additional searches were performed on all databases to identify articles published between May 2020 and January 2022.

### Study selection and data extraction

After initial database searches, duplicates were removed. The remaining records were imported into Excel for screening. The titles and abstracts were screened by the first author according to inclusion and exclusion criteria. Where the abstract was not available or did not provide enough information to guide decision, the full‐text version was retrieved. A random sample of 100 articles was independently screened by a second rater (PW) based on the title and abstract. The agreement rate was 92% (kappa 0.71) and all disagreements were resolved by discussion. Full‐text articles were retrieved and screened by the first author against the eligibility criteria. The second rater independently screened a randomly chosen sample (*n* = 50, 52%) of full‐texts. The agreement rate at this stage was 98% (kappa 0.95). Article key information (reference, diagnosis, control group, sample size, demographics, group characteristics, EF domains, study results) was extracted from the full text versions of articles and stored in an Excel spreadsheet. Second rater (PW) checked data extraction for errors.

### Risk of bias and quality assessment

The Joanna Briggs Institute (JBI) ‐ Critical Appraisal Checklist for Analytical Cross Sectional Studies Moola et al., 2017) used by the first author to assess aspects of study quality and risk of bias in included studies. PW independently rated the quality of all included studies using the same tool (overall agreement rate: 88%, with Kappa for individual items between 0.48 and 0.92 and respective agreement rates 68%–97%). Discrepancies between the two raters were resolved with discussion and a consensus rating was agreed.

### Results synthesis

A narrative synthesis was used to describe the data from included studies. The synthesis was organised by the different domains of EF as outlined in prior literature (e.g. Diamond, 2013): Inhibition, Shifting, WM, Planning, and Composite/Global EF. Attention control (shifting/sustaining attention) was also included as a separate domain given the importance it was given in the EF literature (Garon et al., 2008).

Results were categorised as Significant based on the alpha level of 0.05, unless the paper specified a corrected alpha level. The effect size was categorised as Large (Cohen's d/Hedge's *g* = 0.8, *η* ^2^ = 0.26, partial *η* ^2^ = 0.14), Medium (Cohen's d/Hedge's *g* = 0.5, *η* ^2^ = 0.13, partial *η* ^2^ = 0.06) and Small (Cohen's d/Hedge's *g* = 0.2–0.3, *η* ^2^ = 0.02, partial *η* ^2^ = 0.01), according to prior literature (Cohen, 1992; Hedges, 1981).

## RESULTS

### Study selection

**FIGURE 1:**
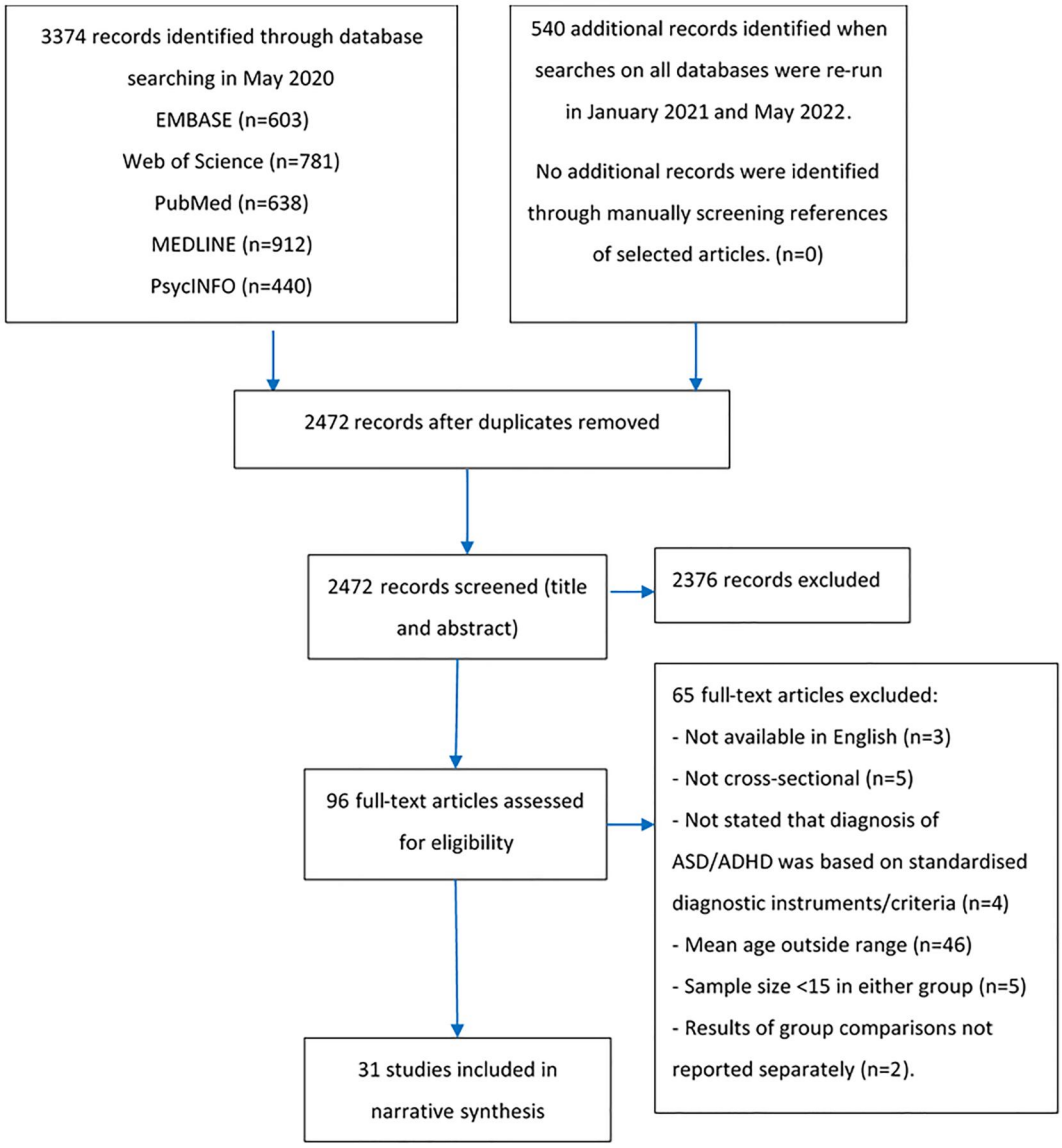
PRISMA flow diagram

A PRISMA flow diagram is presented in Figure 1. The initial search returned 3374 results across databases. Additional searches were conducted to update findings in January 2021 (121 records published between May 2020 and January 2021) and May 2022 (419 records published between January 2021 and January 2022). The references of selected articles were also checked manually after database searches were completed, however this did not yield any additional results. 1308 duplicates were removed after the initial search, 40 duplicates were removed after the second search, and 94 after the third search, thus leaving 2472 articles for Title and Abstract screening. From those, 2376 records were excluded as they did not meet the inclusion criteria.

The full‐texts of 83 articles from the initial database search, four and nine additional articles from the second and third searches respectively were retrieved and assessed for eligibility. The majority of excluded studies at this stage had a sample with a mean age outside the specified range. A total of 31 records met criteria for the review, one of which was retrieved in the second database search, and two from the third search.

### Study characteristics and results

Thirty‐one studies (10 ADHD and 21 ASD studies) met criteria for inclusion. Most control groups consisted of typically developing (TD) children, matched on chronological age (CA) and/or mental age. The majority of studies were conducted in Western countries, particularly the US and UK. Supplementary materials Table S5 provides further details about diagnosis and recruitment for each study.

Table 1 summarises the sample characteristics of ASD and ADHD studies and main findings. Within each diagnostic category (ASD and ADHD), papers are ordered based on their quality score starting from the highest.

**TABLE 1:** Sample characteristics and main findings of included papers

Author (year)	ASD/ADHD	Group size (% boys)	CA in months & IQ Mean (SD)	Control group(s)	Group size (% boys)	CA in months & IQ Mean (SD)	Characteristics matched on	EF domain(s)	Measure(s)	Comparison outcome	Effect size
Gardiner et al. (2017)	ASD	*n* = 24 (83.3%)	**Age**: 66.9 (13.4) **IQ**: 98.9 (15.4)	TD	*n* = 19 (57.9%)	**Age**: 58.5 (15.9) **IQ**: 106.2 (14.4)	CA, maternal education, IQ, gender	WM, planning, inhibition, shifting, global EF	Boxes, Monkey Tower (MT), Boy‐Girl stroop, Preschool CPT, Go/No‐Go, EFCS	**Significant** Parent‐reported global EF (*p* < 0.001) **Not significant** (α level 0.01): Boxes, WM composite (*p*s > 0.20), Go/No‐Go hits, inhibition composite (ps > 0.30), MT (*p*s ≥ 04).^2^	**Large** Global EF *d* = 0.96 **Medium** MT *d* = 0.40 and 0.67 **Small** Boxes *d* = 0.05, Flexibility *d* = 0.25, inhibition *d* = 0.27, WM *d* = 0.33.
Kimhi et al. (2014)	ASD	*n* = 29 (86.2%)	**Age**: 59.5 (11.1) **IQ**: 103.5 (17.2)	TD	*n* = 30 (86.7%)	**Age**: 55.3 (11) **IQ**: 107.6 (14.1)	CA, MA, verbal MA, non‐verbal MA, gender, IQ, maternal education	Set‐shifting, planning	Flexible item selection task,Tower of London	**Significant** ASD group: more shifting errors and lower scores on Tower of London (*p*s < 0.05).	**Medium** Tower of London: *η* ^2^ = 0.07
Griffith et al. (1999)	ASD	*n* = 18 (83.3%)	**Age**: 50.7 (6.7)	DD	*n* = 17 (58.8%)	**Age**: 50.6 (9.2)	CA, verbal and non‐verbal MA, SES	Multi‐component EF tasks	Boxes, A‐not‐B, A‐not‐B with invisible Displacement, Object Retrieval from transparent boxes, Spatial Reversal	**Not significant** All *p*s ≥ 0.075 apart from Spatial Reversal: Autism group had *fewer* failures in maintaining set (*p* = 0.003). Ceiling effects for Object Retrieval.	Not reported.
Valeri et al. (2019)	ASD	*n* = 27 (88.9%)	**Age**: 61.6 (6.4) **Non‐verbal IQ**: 116.6 (12.1)	TD (from BAFE original sample)	*n* = 27 (51.9%)	**Age**: 61.6 (6.3) **Non‐verbal IQ:** 116.6 (10)	CA, non‐verbal IQ	Inhibition, attentional flexibility, set‐shifting, WM	Night/Day, Pattern Making test, Card sort, Spin the Pots	**Significant** Card sort and Night/Day3 (*p*s < 0.05) **Not significant** Pattern Making, Spin the Pots.	**Medium** Card sort shifting *g* = 0.67, Night/Day *g* = 0.69.
Buzzell et al. (2021)	ASD	*n* = 43 (74.4%)	**Age:** 63.1 (4.3) **Non‐verbal IQ:** 106.1 (11.0)	TD	*n* = 24 (58.3%)	**Age:** 63.5 (4.8) **Non‐verbal IQ:** 111.0 (10.2)	CA, gender, non‐verbal IQ, internalising and externalising behaviours	Inhibition	Zoo game (Go/No‐go)	**Significant** Lower behavioural accuracy (*p* < 0.001)	Not reported.
De Lucia et al. (2021)	ASD	*n* = 24 (75%)	**Age:** 64.0 (11.6)	Non‐ASD	*n* = 21 (71.4%)	**Age:** 64.4 (10.6)	CA, gender, ethnicity, SES	Inhibition	Day/Night	**Not significant** No differences (*p* = 0.95)	*d* = 0.02
McEvoy et al. (1993)	ASD	*n* = 17 (58.8%)	**Age**: 60.7 (12.9)	TD	*n* = 16 (62.5%)	**Age**: 37.9 (20)	SES, gender, verbal skill	Multi‐component EF tasks	A‐not‐B (no delay), Delayed response task	**Not Significant** Ceiling effects for both tasks.	Not reported.
Yerys et al. (2007)	ASD	*n* = 18 (83.3%)	**Age**: 34.8 (3.8)	DD and TD (TD1 and TD2)	DD: *n* = 18 (55.6%) TD1: *n* = 18 (44.4%) TD2: *n* = 18 (33.3%)	**Age**: DD: 35.5 (5.6) TD1: 22.2 (4.5) TD2: 32.6 (4.6)	MA (TD1), CA (TD2)	Multi‐component EF tasks	Windows task, Spatial Reversal, A‐not‐B	**Not Significant** No differences in the expected direction. TD1 made more perseverative errors than ASD on Spatial Reversal (*p* = 0.04) and more errors after an error on A‐not‐B (*p* = 0.05).	Not reported.
Jahromi et al. (2013)	ASD	*n* = 20 (90% in whole sample of 40)	**Age**: 59 (11.5)	TD	*n* = 20	**Age**: 50.2 (11.1)	MA, gender, expressive and receptive language	Inhibition	Day/Night, BRIEF‐P	**Significant** Inhibitory self‐control index (BRIEF‐P) and inhibition composite (*p*s < 0.01) **Not significant** Day/Night task.	**Large** Inhibition composite: Partial *η* ^2^ =0.17, BRIEF‐P inhibitory self‐control: 0.23 **Small** Day/Night partial *η* ^2^ = 0.04
Pellicano et al. (2006)	ASD	*n* = 40 (87.5%)	**Age**: 67.2 (10.9) **Verbal IQ**: 101.2 (11) **Non‐verbal IQ:** 113.6 (14.1)	TD	*n* = 40 (77.5%)	**Age**: 65.7 (11.5) **Verbal IQ**: 103.3 (9.9) **Non‐verbal IQ:** 112.5 (14.5)	CA, verbal IQ, non‐verbal IQ, gender	Inhibition, planning, set‐shifting	Luria's hand‐game, Mazes, Tower of London, Teddy Bear card‐sorting task	**Significant** Luria's hand‐game (*p* < 0.005), Tower of London^4^ and Teddy Bear (*p*s < 0.001). **Not significant** Mazes task.	**Medium‐large** Luria's hand‐game: *η* ^2^ = 0.10, Tower of London: Partial *η* ^2^ = 0.20, Teddy Bear: *η* ^2^ = 0.16
Pellicano (2007)	ASD	*n* = 30 (83.3%)	**Age**: 67.6 (11.7) **Verbal IQ**: 100 (10.6) **Non‐verbal IQ**: 113.9 (13.7)	TD	*n* = 40 (77.5%)	**Age**: 65.7 (11.5) **Verbal IQ**: 103.3 (9.9) **Non‐verbal IQ:** 112.5 (14.5)	CA, verbal IQ, non‐verbal IQ	Planning, set‐shifting, inhibition	Mazes task, Tower of London, Teddy Bear card‐sorting task, Luria's hand‐game	**Significant** Luria's hand‐game (*p* < 0.05), Tower of London (*p* < 0.001), and Teddy bear (*p* < 0.005). **Not significant** Mazes task.	**Medium‐large** Luria's hand‐game: η2 = 0.07, Tower of London: η2 = 0.16, set‐shifting: η2 = 0.12
Garon et al. (2018)	ASD	*n* = 34 (79.4%)	**Age**: 54.8 (11.1) **IQ**: 81.9 (25.3)	TD	*n* = 255 (57.6%)	**Age**: 43 (12.1) **IQ**: 99.6 (16)	MA	WM, inhibition, set‐shifting	Hide and seek, Tricky Box, Flap book	**Significant** All *p*s < 0.01. (Inhibition and shifting *p* < 0.001).^5^ Pattern of results the same in older and younger pre‐schoolers.	**Medium** Inhibition: *d* = 0.67 Shifting: *d* = 0.52 WM: *d* = 0.45
Smithson et al. (2013)	ASD	*n* = 44 (77.3%)	**Age**: 53 (9.5) **Verbal IQ**: 93.1 (13) based on a sub‐sample (*n* = 39)	TD (from BRIEF‐P original sample)	*n* = 44 (82.1% boys based on a sub‐sample *n* = 39)	**Age**: 52.8 (9.2) for sub‐sample (*n* = 39) **Verbal IQ**: assumed to be average (100)	CA, gender. Separate analysis (ASD *n* = 29) with groups matched on verbal IQ and non‐verbal IQ.	Inhibition, shifting, WM, planning, global EF	BRIEF‐P parent ratings	**Significant** Group effects (*p*s < 0.01) on all scales, indices and GEC, also when matched on verbal and nonverbal IQ.	**Large** Partial η^2^: 0.33‐0.37 (scales) and 0.31‐0.36 (indices). GEC: *d* = 1.46–1.57.
Dawson et al. (2002)	ASD	*n* = 72 (83.3%)	**Age**: 43.5 (4.3)	DD and TD	DD: *n* = 34 (52.9%) TD: *n* = 39 (76.9%)	**Age**:	MA, SES, ethnicity	Multi‐component EF tasks	A‐not‐B, Spatial Reversal, Delayed non‐matching to sample, Object Discrimination Reversal	**Not significant** Floor effect for 90% of children on spatial Reversal.	Not reported.
						DD: 44.8 (5.3) TD: 27.1 (8.9)					
Zhao et al. (2019)	ASD	*n* = 47 (87.2%)	**Age**: 63 (14) **Non‐verbal IQ** (raw score): 22.9 (7.4)	TD	*n* = 51 (60.8%)	**Age**: 57 (9) **Non‐verbal IQ** (raw score): 22 (3.3)	Non‐verbal IQ	Set‐shifting	Dimensional change card sorting (DCCS)	**Significant** Group differences *p* = 0.008.	Not reported. Calculated: *d* = 0.58 (**Medium**)^6^
Pellicano et al. (2017)	ASD	*n* = 30 (90%)	**Age**: 53.3 (12.2) **Non‐verbal IQ**: 101.6 (15) **Verbal IQ**: 96.5 (15.5)	TD	*n* = 30 (66.7%)	**Age**: 53 (10.5) **Non‐verbal IQ:** 102.3 (12.6) **Verbal IQ**: 101.9 (11)	CA, performance IQ and verbal IQ.	Set‐shifting, WM	DCCS, Corsi Blocks	**Significant** Group differences on both tasks, *p*s ≤ 0.001.	**Large** Corsi Blocks partial *η* ^2^ = 0.35 and DCCS: Partial *η* ^2^ = 0.33
Smith et al. (2019)	ASD	*n* = 29 (96.6%)	**Age**: 27.4 (4.5) **IQ**: 64.8 (12.8)	TD	*n* = 30 (43.3%)	**Age**: 27.3 (5.8) **IQ**: 107.4 (15.6)	CA	Attention shifting	Novel eye‐tracking visual search paradigm with switching targets.	**Not significant** No group differences on hit rates, fixation durations or fixation latencies.	**Small** Partial η^2^ range: 0.001 (Fixation durations) ‐ 0.019 (Hit rates).
Fanning et al. (2018)	ASD	*n* = 26 (86.6%)	**Age**: 45.5 (9.5) **IQ**: 60.1 (22.3)	TD and Williams syndrome	TD: *n* = 19 (68.4%), WS: *n* = 18 (55.6%)	**Age**: TD: 50.9 (12.4) WS: 49.8 (16.3) **IQ:** TD: 104.4 (14.2) WS: 56.8 (15.8)	All matched on CA. The ASD and WS groups were matched on total IQ, verbal IQ, and non‐verbal IQ.	Spatial WM	Novel eye‐tracking paradigm based on the A‐Not‐B task.	**Not significant** Total looking time towards target location did not differ across groups.	**Small** Partial η^2^: 0.04 (for interaction between group and location)
Leekam et al. (2000)	ASD	*n* = 18 gender not reported	**Age**: 52.3 (10.9) **Non‐verbal IQ**: 77.7 (38.9)	DD	*n* = 19 gender not reported	**Age**: 53.6 (6.8) **Non‐verbal IQ:** 67.5 (34.3)	Non‐verbal ability	Attention shifting	Attention switching between target and peripheral targets on computer screen (measured in head turns)	**Not significant** No group differences on proportion of correct responses. ASD group gave faster responses than DD (*p*s < 0.05).	Not reported.
Rutherford and Rogers (2003)	ASD	*n* = 28 gender not reported	**Age**: 33.9 (3.5)	Developmental Disorders^7^ (DD) and TD	DD: *n* = 24 TD: *n* = 26 gender not reported	**Age**: DD: 34.8 (6.7) TD: 19.5 (4.7)	MA (overall and non‐verbal)	Multi‐component EF tasks	Spatial Reversal, Generativity (designed by authors)	**Not significant** No differences between ASD and either control group.	Not reported.
Stahl and Pry (2002)	ASD	*n* = 15 (86.7%)	**Age**: 60.7 (11.2)	TD	*n* = 21 (52.4%)	**Age**: 25.2 (3)	No matching.	Set‐shifting	Multi‐step multi‐location task (designed by authors)	**Not significant** No group differences on correct switches. Ceiling scores for one‐third of children in ASD group and 1/7 of the TD group.	Not reported.
**ADHD studies**											
Çak et al. (2017)	ADHD	*n* = 21 (85.7%)	**Age**: 58.1 (8.3) **IQ**: 98.1 (19.5)	TD (from BRIEF‐P original sample)	*n* = 52 (65.4%)	**Age**: 56.9 (9.1) **IQ**: 109.5 (33.8)	CA, gender, SES, IQ, parental education and age, maternal occupation, number of siblings, developmental milestones, perinatal complications	Inhibition, sustained attention, shifting, WM, planning, global EF	K‐CPT, BRIEF‐P	**Significant** All BRIEF‐P scales^8^ and GEC: *p*s < 0.001. K‐CPT omissions, commissions, hit reaction time SE/inter‐stimulus interval change, variability (*p*s: 008‐0.039). Group effect on K‐CPT *p* = 0.003.*^5^	**Large** BRIEF‐P range of d: 3.38 (shift) – 3.95 (WM). GEC *d* = 4.66. K‐CPT significant results d: 0.72 – 0.95. Effect of group η^2^: 0.25 (BRIEF‐P), 0.75 (K‐PCT).
Schneider et al. (2020)	ADHD	*n* = 49 (59.2%)	**Age**: 60 (7.2) **IQ**: 108.4 (11.6)	TD	*n* = 35 (51.4%)	**Age**: 58.8 (6.0) **IQ**: 109.7 (13.2)	CA, SES, IQ, race, gender, core language skills	Inhibition, shifting, WM, planning	BRIEF‐P (parent and teacher ratings)	**Significant** Group effect on all scales (*p*s < 0.001). Parents reported more impairments in WM and planning than teachers (*p*s < 0.01).	**Large** WM and planning partial η^2^ for parents: 0.59 and 0.51, teachers: 0.25 and 0.20.
Lacerda et al. (2020)	ADHD^9^	*n* = 24 (50%)	**Age**: 67 (11) **IQ**: 69.9 (15.9)	Born very pre‐mature and/or with very low birth weight (No ADHD)	*n* = 55 (47.3%)	**Age**: 65.5 (8.6) **IQ**: 76.6 (17.2)	CA, gender, IQ, SES, gestational age, anxiety	Inhibition, sustained Attention, shifting, WM, planning	K‐CPT 2, BRIEF‐P (parent ratings)	**Not significant** No group differences on any measure.	**Small** Partial η^2^ range: 0.001 (BRIEF‐P Flexibility, Commission errors on K‐CPT) – 0.046 (BRIEF‐P inhibitory self‐control).
Schneider et al. (2016)	ADHD	*n* = 33 (69.7%)	**Age**: 64.4 (10.8) **Verbal IQ**: 110 (9.6)	TD	*n* = 31 (58.1%)	**Age**: 68.2 (11.8) **Verbal IQ**: 118 (10.8)	CA, SES, gender	Sustained Attention, WM, inhibition/Motor inhibition, global EF	ACPT‐P, CANTAB Auditory and spatial WM, Stop Signal, NEPSY‐II Statue, Conflicting motor response, BRIEF‐P	**Significant** Global EF, WM and inhibition tasks, remained significant after controlling for sleep (apart from Conflicting motor response). **Not significant** ACPT‐P (variability *p* = 0.74, mean reaction time *p* = 0.028).	**Large** Global EF: *η* ^2^ = 0.35 (parent) and 0.37 (teacher) **Medium** Range of η^2^: 0.082 (Conflicting motor response) ‐ 0.13 (spatial WM). **Small‐medium** ACPT‐P η^2^: 0.002, 0.078
Zhang et al. (2018)	ADHD	*n* = 163 (82.2%)	**Age**: 59.1 (7.2) **IQ**: 104.6 (17.9)	TD	*n* = 63 (71.4%)	**Age**: 59.7 (5.3) **IQ**: 115 (12.6)	CA, gender	Inhibition, shifting, WM, planning, global EF motor inhibition	BRIEF‐P parent ratings NEPSY‐II Statue	**Significant** All group differences on BRIEF‐P: *p*s < 0.001 apart from shifting: *p* = 0.039. Statue: *p* = 0.001	**Large** WM *η* ^2^ = 0.28, BRIEF‐P inhibition *η* ^2^ = 0.40, GEC *η* ^2^ = 0.32. **Small‐medium** Shifting *η* ^2^ = 0.02, planning *η* ^2^ = 0.20. Statue *η* ^2^ = 0.10
Mariani et al. (1997)	ADHD	*n* = 34 (100%)	**Age**: 60.1 (7.5) **IQ**: 107.8 (13.2)	TD	*n* = 30 (100%)	**Age**: 61 (6.3) **IQ**: 114.5 (10.2)	CA, social class, abstract reasoning IQ, school experience	WM (auditory and spatial), planning, set‐shifting, sustained Attention, inhibition/impulse control	K‐ABC Number Recall and Spatial Memory, Porteus Mazes, Colour form test, CPT	**Significant** WM (auditory: *p* = 0.024, spatial: *p* = 0.001), planning (*p* = 0.006), Attention (CPT number correct: *p* = 0.004). **Not significant** Colour form, CPT commission errors.	Not reported.
Mahone and Hoffman (2007)	ADHD	*n* = 25 (80%)	**Age**: 58.3 (10) **Verbal IQ**: 97 (14.2)	TD (from BRIEF‐P original sample)	*n* = 25 (80%)	**Age**: 58.2 (10) **Verbal IQ** assumed to be average	CA, gender, race, maternal education	Inhibition, shifting, WM, planning, global EF	BRIEF‐P parent ratings	**Significant** For all scales and indices of BRIEF‐P, and the GEC (*p* < 0.01).^10^	**Large** BRIEF‐P scales *d* range: 0.8 (shifting) ‐ 2.4 (WM). GEC *d* = 2.0
Sjöwall and Thorell (2019)	ADHD	*n* = 52 (77%)	**Age**: 71 (6.7)	TD	*n* = 72 (56%)	**Age**: 66.2 (8.6)	No matching (controlled for parental education in the analysis)	Inhibition, WM, global EF	Go‐No‐Go, Backward Digit span, Find the Phone, CHEXI (teacher ratings)	**Significant** Find the Phone (*p* < 0.05). All other tasks and global EF (*p*s < 0.001).	**Large** CHEXI *η* ^2^ = 0.76. **Medium** η^2^ = 0.10 (Go‐No‐Go) −0.13 (Backward Digit). **Small** η^2^ = 0.06 (Find the Phone).
Dalen et al. (2004)	ADHD	*n* = 19 gender not reported	**Age**: 39.4 **IQ**: 99.9	TD	*n* = 19 gender not reported	**Age**: 38.9 **IQ**: 100.8	CA, gender, IQ	Inhibition, set‐shifting	Go‐No‐Go inhibition, set shifting‐modified Weigl block sorting task	**Significant** Go‐No‐Go inhibition (*p* < 0.01). **Not significant** Set‐shifting on block sorting task.	Not reported.
Schoemaker et al. (2012)	ADHD	*n* = 61 (80.3%)	**Age**: 55.2 (7.4) **IQ**: 101.3 (12)	DBD TD	DBD: *n* = 33 (81.8%) TD: *n* = 56 (69.6%)	**Age** DBD: 51.9 (8.3) TD: 55.7 (7.2) **IQ** DBD: 101.9 (10.9) TD: 111.7 (10.3)	CA, gender	Inhibition, WM	Go‐No‐Go, Shape School – Inhibition, Nine Boxes, Delayed Alternation	**Significant** Overall EF and shape school (*p*s < 0.001), Go‐No‐Go and delayed Alternation (*p*s < 0.01). **Not significant** Nine Boxes.	Not reported.

Abbreviations: ACPT‐P, Auditory Continuous Performance Task‐Preschool; ADI‐R, Autism Diagnostic Interview–Revised (Lord et al., 1994); ADOS, Autism Diagnostic Observation Schedule (Lord et al., 2000, Lord; Luyster, et al., 2012, Lord; Rutter, et al., 2012); BRIEF‐P, Behaviour Rating Inventory of Executive Function‐Preschool Version (Gioia et al., 2002); CA, Chronological Age; CANTAB, Cambridge Neuropsychological Test Automated Battery (CeNes Cognition, 1996); CHEXI, Childhood Executive Functioning Inventory (Thorell & Nyberg, 2008); CPRS‐R, Conners' Parent Rating Scale – Revised (Conners, 1997); CPT, Continuous Performance Test; DBD, Disruptive Behaviour Disorders; DCCS, Dimensional Change Card Sort; DD, Developmental Delay; EFCS, Executive Function Content Scale (BASC‐2; Reynolds & Kamphaus, 2004); GEC, Global Executive Composite; IQ, Intelligence Quotient; K‐CPT, Conners' Kiddie Continuous Performance Test; MA, Mental Age; NEPSY, Developmental NEuroPSYchological Assessment (Korkman et al., 2007); SE, Standard Error; SES, Socioeconomic Status; TD, Typically Developing; WM, Working Memory; WS, Williams Syndrome; Numbers in the Group size and CA/IQ columns were rounded to 1 decimal place, For a full list of laboratory‐based tasks with their citations see Supplementary Materials Table S6.

### Quality assessment

Two studies (Schneider et al., 2020; Çak et al., 2017) achieved a perfect quality score, as they adequately addressed all elements of quality assessed by the rating scale. Twelve studies were assigned a good quality score (5/7‐6/7), ten received a fair quality rating of 4/7, with the seven remaining studies receiving lower scores indicating poor quality. The elements that commonly contributed to lower quality ratings included unclear inclusion criteria and lack of identification and control of potential confounders. Moreover, a substantial number of studies (14 in total) used tasks with unclear or untested validity and reliability. Supplementary Materials Table S7 presents a breakdown of the ratings for each study and further information regarding the quality assessment process.

### Synthesis of results

The results below are summarised separately for ASD and ADHD under each EF domain. The results of tasks designed to tap multiple EF domains are summarised separately.

### Inhibition

#### Autism Spectrum Disorder

Six out of the nine ASD studies that measured Inhibition found the ASD group to be significantly more impaired than the TD group, and reported medium effect sizes for laboratory tasks, and large effect sizes for informant ratings (apart from Buzzell et al., 2021 who did not report an effect size). Gardiner et al. (2017), DeLucia et al. (2021) and Jahromi et al. (2013) did not find any significant group differences on response inhibition (Go‐No‐Go) and interference control tasks (all three used Stroop‐like paradigms), though Jahromi et al. found significant, large group differences on the BRIEF‐P. Only Gardiner et al. and DeLucia et al. measured and controlled for SES. Gardiner et al. and Valeri et al. (2019), who also used a Stroop task, found an association between Inhibition scores and ASD severity scores. Garon et al. (2018) found Inhibition, measured by a similar interference control task, to be the best predictor of group (ASD/TD) membership, followed by Shifting and then WM.

#### ADHD

ADHD studies consistently showed significant Inhibition impairments in the ADHD group, with two studies reporting an association between Inhibition and ADHD symptoms. Medium‐large Inhibition impairments were found on different types of inhibition tasks (e.g. Stop Signal, Statue, Go‐No‐Go) as well as informant ratings, though three studies out of the total of ten did not report effect sizes. Lacerda et al. (2020) did not find a significant group difference in informant ratings and measures of response inhibition, though their sample consisted of children that were born very premature/with very low birth weight. The authors suggest that the absence of differences could be attributed to the comparison group already being very cognitively impaired.

### Shifting

#### Autism Spectrum Disorder

Seven studies that used set‐shifting tasks, such as card sort and flexible item selection, and one study that used informant ratings found significant, medium‐large impairments for the ASD group compared to a TD group. Only one of these studies (Kimhi et al., 2014) controlled for SES. Stahl and Pry (2002) did not find a significant group difference, though this study was assigned a low quality score. Shifting was significantly associated with ASD severity and the best predictor of group (ASD/TD) membership after Inhibition in Garon et al. (2018).

#### ADHD

ADHD study findings on Shifting were less consistent. Four studies found Shifting to be significantly impaired in the ADHD group compared to the TD group when measured by the BRIEF‐P, though its effect size was the smallest compared to the other EF domains reported, and ranged from small to large. Mahone and Hoffman (2007) additionally found Flexibility on the BRIEF‐P to be significantly correlated with ADHD symptoms. Studies did not find significant differences when the ADHD group was compared to a group of children born prematurely/with low birth weight (Lacerda et al., 2020) or where set‐shifting tasks were used (Mariani and Barkley (1997); Dalen et al., 2004).

### Working memory

#### Autism Spectrum Disorder

Findings of the six studies that specifically measured WM using different laboratory tasks are mixed. Half the studies did not find a significant difference between ASD and control group; those studies matched groups on IQ, CA and maternal education. The other three studies found significant, medium‐large WM impairments on other laboratory tasks and also on informant ratings. None of the studies found a significant association between WM and ASD symptoms.

#### ADHD

All ADHD studies with the exception of Dalen et al. (2004) measured WM, and most of them found a significant group difference. Four studies reported significant differences on informant‐rated WM, controlled for different cofounders and reported mostly large effect sizes (though two studies that did not report effect size). Four studies measured WM using different laboratory tasks; three of those reported significant group differences. Lacerda et al. (2020) found no group differences on the BRIEF‐P in their sample of children born prematurely/with low birth weight. Similarly to ASD studies, no study found a significant association between WM and ADHD symptoms.

### Planning

#### Autism Spectrum Disorder

Studies measuring Planning in ASD report mixed findings, largely dependent on the measure used. Tower of London yielded significant group differences with medium‐large effect sizes in three studies. However, where an age‐adapted tower task and a simple planning task were used, no group differences were found. Scores on tower tasks were associated with autism severity scores (Gardiner et al., 2017) and were predictive of group membership (Pellicano et al., 2006). Planning was also found to be significantly worse in the ASD group when rated by parents (Smithson et al., 2013).

#### ADHD

Findings with regards to planning consistently show significant, mainly large impairments for the ADHD group when compared with a TD group on informant ratings and on a simple planning task. One study out of the five that found planning impairments reported a medium effect size, and one study did not report effect sizes. Only Lacerda et al. (2020) did not find a significant difference in parent‐rated planning in their sample of prematurely‐born preschoolers. No study found any significant associations between Planning and ADHD symptoms.

### Attentional control

#### Autism Spectrum Disorder

Only three ASD studies explicitly measured attentional control, and specifically attention shifting. Valeri et al. (2019) used a previously validated task, whereas Smith et al. (2019) and Leekam et al. (2000) used novel tasks requiring attention switching between visual targets. The studies failed to find any significant differences between ASD and either TD or developmentally delayed children.

#### ADHD

Four ADHD studies measured sustained attention using a type of Continuous Performance Test (CPT), two of which found the ADHD group to be significantly more impaired than the TD group. Çak et al. (2017) also reported a large effect size for overall K‐CPT performance and a moderate correlation between K‐CPT inattention and ADHD symptoms. Schneider et al. (2016) did not find any group differences, though their sample was slightly older and different components of the CPT were assessed. Lacerda et al. (2020), did not find any group differences in K‐CPT scores in their sample of children born prematurely, in line with the lack of group differences reported for the other domains.

### Multi‐component tasks and global EF

#### Autism Spectrum Disorder

Five ASD studies used multi‐component EF tasks, which usually require maintenance of information over a delay, updating this with new information, inhibiting a pre‐potent response, and switching to a different response or search strategy. In most cases, those were administered to younger preschoolers (below the age of 5). Studies failed to find any significant differences between the ASD group and either a TD or a developmentally delayed control group. Yerys et al. (2007) and Griffith et al. (1999) even found that the control groups made more errors than the ASD group. Studies that measured Global EF through informant report reported large group differences, even after controlling for age, maternal education, IQ and gender.

#### ADHD

Four ADHD studies measured Global EF through informant ratings and reported significant, large impairments for the ADHD group. Most studies apart from Sjöwall and Thorell (2019) controlled for CA, SES and gender.

Table 2 below summarises the study findings for the different EF domains in preschool ASD and ADHD.

**TABLE 2:** Summary of executive functioning (EF) impairments in preschool autism spectrum disorder (ASD) and ADHD

EF domain	ASD	ADHD
**Inhibition**	**Mostly impaired** ‐informant ratings and *some* laboratory tasks	**Impaired** ‐informant ratings and laboratory tasks
**Shifting**	**Impaired** ‐informant ratings and laboratory tasks	**Mixed findings**: Not impaired on laboratory tasks, impaired on informant ratings
**Working memory**	**Mixed findings**: Impaired only in half the studies	**Impaired** ‐informant ratings and laboratory tasks
**Planning**	**Mixed findings**: Impaired on parent ratings and higher order planning tasks, not impaired on simple/age‐adapted planning tasks	**Impaired** ‐simple planning task and informant ratings
**Attentional control**	**Not impaired** ‐laboratory tasks measuring Attention shifting^a^	**Mixed findings** for sustained Attention on laboratory tasks
**Global EF**	**Impaired** ‐informant ratings	**Impaired** ‐informant ratings

^a^ Only three studies, some issues with study quality.

## DISCUSSION

### Summary of findings

There was agreement among studies that both ASD and ADHD preschoolers are markedly impaired in global EF, when that was rated by parents or teachers. This reported impairment was robust and not dependent on factors like parental education, gender and IQ. When considering individual domains, Inhibition was significantly impaired in both ASD and ADHD in comparison to a TD group, though more consistently in ADHD. Shifting was more consistently impaired in ASD preschoolers, as ADHD preschoolers were impaired on informant ratings but not laboratory tasks. Working Memory yielded mixed findings in ASD, while most ADHD studies reported large impairments on tasks as well as informant ratings. ASD preschoolers were impaired in Planning compared to TD children on informant ratings and the Tower of London task, but not on simplified or age‐adapted tasks; there was a clear Planning impairment in ADHD, mainly on informant ratings but also a simple planning task. Different types of attention were measured in ASD and ADHD studies. ASD studies failed to find any group differences in attention shifting, though the studies were generally of lower quality. ADHD studies on sustained attention yielded mixed findings. Taken together, EF difficulties are prevalent in both preschoolers with ASD and ADHD, but may show slightly distinct profiles in the two conditions.

### Links to prior literature

The findings of this review are similar to Visser et al. (2016), who reviewed younger and subclinical samples, and Craig et al. (2016), who reviewed studies of children and adolescents. Both reported more apparent Inhibition impairments in ADHD, and found Shifting to be more consistently impaired in ASD. Shifting in preschool ADHD yielded the lowest effect size compared to other domains, in line with findings of meta‐analyses of young children with current or later‐emerging ADHD and children at elevated likelihood for ADHD (Pauli‐Pott & Becker, 2011; Shephard et al., 2021). Moderate inhibition impairments were also noted in older children and adults with ASD (Demetriou et al., 2018). Moreover, robust Planning and WM impairments in ADHD were reported for older children and adolescents with ADHD (Willcutt et al., 2005), and are highlighted as potential early‐life (0–5 years) precursors for ADHD (Shephard et al., 2021). Therefore, findings in studies of younger preschoolers, older children and adolescents/adults appear to be broadly consistent, indicating at least some stability in EF profiles over time in the two conditions.

Most studies that measured specific EF domains in the current review recruited older preschoolers (over the age of four). Studies that recruited younger preschoolers typically used multi‐component EF tasks and failed to find any significant group differences. This was mostly the case in ASD studies, where some authors concluded that EF impairments do not emerge until later in life and are only secondary to ASD (Dawson et al., 2002; Griffith et al., 1999; Yerys et al., 2007). Nevertheless, some of those studies reported ceiling and floor effects across groups, while the reliability of some of the multi‐component tasks has been questioned (e.g. Griffith et al., 1999; Yerys et al., 2007). This raises the question of whether these tasks are appropriate or sensitive enough to detect executive dysfunction in this age group, as some, such as the A‐not‐B task, had been originally developed for infants, and others for non‐human primates (e.g. Boxes; Petrides, 1995). Nonetheless, it may also be the case that EF difficulties emerge or consolidate later in the preschool period.

### Limitations

Only published papers available in English were included in this review, which might have introduced publication bias. Potentially helpful insights from unpublished material, or studies published in other languages and low‐income countries, might have been missed. The majority of ADHD studies and a third of ASD studies excluded participants with a low IQ, thus it is possible that the review findings may not extend across the whole range of ability seen in young children with ASD and ADHD. However, it is hard to assess representativeness as nine studies did not include any measure of cognitive ability. ADHD studies included slightly older preschoolers, which was expected, as diagnostic classification systems advise caution in diagnosing ADHD in preschool children (e.g. ICD‐10; World Health Organization, 1993). This could potentially mean that ADHD studies recruited samples that form a less representative subset of the ADHD population in comparison to the ASD studies, for example, children with more severe ADHD symptoms or behaviours which would make them more likely to be identified and diagnosed in early childhood. Notwithstanding the fact that ASD is often identified earlier than ADHD, this could also be the case for children with ASD identified during the preschool period; thus the findings around the preschool EF profiles described in this review may not apply to all individuals with a lifetime diagnosis of ASD or ADHD. Additionally, ASD studies did not measure the co‐occurrence of ADHD symptoms (or vice‐versa), despite a substantial proportion of children presenting with comorbid ADHD and ASD (Rommelse et al., 2010a). Therefore, the possibility of neurodevelopmental comorbidity contributing to the overlap in EF profiles cannot be eliminated. There is also a lack of studies directly comparing children with ASD and those with ADHD on the same task/paradigm.

Most ASD studies used different laboratory tasks, while most ADHD studies used informant report (more commonly the BRIEF‐P). It is possible that the consistency in assessment methodology might account for the large effects and the relatively higher agreement among ADHD studies in comparison to ASD studies. The only discrepancies observed in the ADHD studies were for domains assessed through laboratory tests (e.g., Shifting). This discrepancy in findings corresponding to laboratory versus informant based methods was reported in the broad EF/self‐regulation field (e.g. Eisenberg et al., 2019) but also in relevant reviews, such as Demetriou et al. (2018), who noted that studies using informant report tended to show more marked EF deficits compared to those using neuropsychological tasks. Laboratory tasks are conducted in controlled environments under optimal, highly structured conditions, which might enable children with neurodevelopmental conditions to perform at their maximum capacity and thus within the range of typically‐developing children (Toplak et al., 2013), potentially explaining the null findings in some of these studies. Conversely, informant ratings might be influenced by caregivers' or teachers' views of the child as generally “problematic”, which might account for the high and overlapping impairments found across domains in informant ratings, but not in task performance (Hendry et al., 2016; Sjöwall & Thorell, 2019).

Lastly, another limitation that might explain some of the mixed findings is the task impurity problem, as EF tasks often require the coordination of lower‐order skills (e.g. motor skills) and may tap multiple EFs and other cognitive processes, such as attention, short‐term memory, language ability, spatial/visual processing and processing speed. Given the differential impairments found in early and middle childhood (Craig et al., 2016; Visser et al., 2016), the findings were organised into EF domains for comparison purposes and to provide as clear a picture of EF profiles as possible. Although an attempt was made to separate the more composite tasks from those explicitly used to measure a specific EF domain, the latter may still not accurately reflect “pure” EFs but rather a combination of target EF and other EF and non‐EF component effects (Hendry et al., 2016; Snyder et al., 2015). Therefore, the variance attributable to the target EF may be smaller than what specific‐EF tasks assume, and may vary across tasks, which could explain why some tasks yielded significant group differences and others did not.

### Future directions and implications

Future studies of EF in ASD and ADHD should employ mixed methodology when assessing EF and balance the use of informant‐based methods with that of laboratory‐based tasks. There is a clear need for more reliable, ecologically‐valid and developmentally appropriate EF tasks for younger preschoolers. This is particularly important when assessing working memory and attentional control, as the tasks used in the reviewed studies produced mixed findings, and some were not previously validated. Eye‐tracking and touch‐screen paradigms are promising tools as they can measure cognitive and attentional functions in young children in a reliable way, potentially partialling out confounding factors, like social motivation, as they involve fewer interactions with the researcher and rely less on the child's language and motor skills, which are inherent in other test procedures and may be impaired in children with neurodevelopmental conditions (Hendry et al., 2016). In order to control for other confounding cognitive processes, studies should also carefully select targeted measures that place higher demands on the target EF compared to other processes, and statistically combine several of those measures to derive a latent variable, which might be a “purer” measure of the target EF (see Snyder et al., 2015). Moreover, future studies should pay more attention not only to ADHD‐ASD co‐occurrence, but also to socioeconomic differences between the clinical and TD groups, as those have been found to be associated to both neurodevelopmental conditions and poorer EF outcomes (see section on quality assessment in Supplementary Materials for more details).

Findings point to EF as a shared early process on the pathways to ASD and ADHD, though the specific executive domains implicated may be quantitatively different (e.g. may differ in degree and consistency) for each condition. This further highlights the importance of assessing and addressing EF in preschoolers with ASD and ADHD. Shifting in ASD and inhibition in ADHD are promising early intervention targets, as they may form the basis of later‐emerging higher‐order functions (Diamond, 2013). Working memory could also be an important intervention target in ADHD. Cognitive training utilising age‐appropriate activities has demonstrated promising preventative effects when delivered during the early preschool period (Wass, 2015), while improvements from training can generalise beyond the specific EFs targeted (Scionti et al., 2020), suggesting a degree of interconnectedness between different EFs, which could render interventions at this developmental stage particularly beneficial.

## CONCLUSIONS

Based on the studies reviewed, informant reports provide robust evidence of a global EF impairment in both ASD and ADHD from the fourth year of life. EF profiles in preschool ASD and ADHD overlap to some extent, but there are indications of differences in the consistency of domain‐specific impairments: Shifting seems to be more consistently impaired in ASD compared to ADHD, while WM, Planning and Inhibition are more consistently impaired in ADHD. The EF profile of ADHD preschoolers seems to comprise robust impairments in a larger number of EF domains in comparison to that of ASD preschoolers. However, due to methodological limitations and the different methods of measuring EF, it is still unclear whether these differences are robust enough to reflect different underlying EF profiles and distinct developmental processes in the pathways to ASD and ADHD. Further research on early executive impairments in neurodevelopmental conditions may identify fruitful targets for intervention at a very crucial stage in development.

## AUTHOR CONTRIBUTIONS

**Marina Christoforou**: Conceptualization; Methodology; Resources; Software; Writing – original draft; Writing – review & editing. **Emily J. H. Jones**: Methodology; Supervision; Writing – original draft; Writing – review & editing. **Philippa White**: Validation; Writing – review & editing. **Tony Charman**: Conceptualization; Supervision; Writing – review & editing.

## CONFLICTS OF INTEREST

Emily J. H. Jones is a Joint Editor for JCPP Advances. Tony Charman has served as a paid consultant to F. Hoffmann‐La Roche Ltd. and Servier; and has received royalties from Sage Publications and Guilford Publications. The remaining authors have declared that they have no competing or potential conflicts of interest.

## ETHICAL CONSIDERATIONS

Not applicable.

## Supporting information

Supporting Information S1

## Data Availability

Data sharing is not applicable to this article as no new data were created or analyzed in this study.

## ENDNOTES

^1^
This was decided to avoid including studies with very low statistical power, which was not evaluated in the quality assessment.

^2^
Inhibition composite and planning (Monkey Tower‐highest level achieved) associated with higher autism severity scores in ASD group.

^4^
Significantly predicted group (TD/ASD) membership, *p* < 0.001.

^5^
Best predictors of group membership: complex Inhibition, followed by simple Inhibition, complex Shifting and simple Shifting. Shifting was significantly correlated with ADOS severity scores, but not ADI‐R total scores.

^6^
Not provided by authors but calculated from data provided.

^7^
Group consisted of children with other developmental disorders (e.g. Down Syndrome)

^8^
The CPRS‐R/S ADHD index was strongly correlated with inhibition and related indices (BRIEF‐P) and was moderately correlated with K‐CPT inattention (omission rates, Hit reaction time Standard Error/inter‐stimulus interval change, variability).

^9^
Children in the ADHD group were born very pre‐mature and/or with very low birth weight, like those in the control group, but were also given an ADHD diagnosis.

^10^
ADHD symptoms were significantly correlated with the BRIEF‐P Inhibitory Self‐Control Index and the GEC, and three of the four CPRS‐R scales were significantly correlated with the Flexibility Index.
