# Supplementary material for: Executive function profiles of preschool children with autism spectrum disorder and attention‐deficit/hyperactivity disorder: A systematic review
Source: JCPP Adv. 2023 Jan 7;3(1):e12123. doi: 10.1002/jcv2.12123 (PMC10241451; doi:10.1002/jcv2.12123)
Supplement: Supplementary file 1 — Supporting Information S1 [file JCV2-3-e12123-s001.docx]

**Supplementary materials**

Table S1: Electronic database search terms optimised for EMBASE……………………………….….2

Table S2: Electronic database search terms optimised for MEDLINE & PubMed…….………….3

Table S3: Electronic database search terms optimised for PsycINFO………………………………….4

Table S4: Electronic database search optimised for Web of Science…………………….…………….5

Table S5: Recruitment and diagnosis details for included studies……………………………………….6

Table S6: Laboratory-based tasks in included studies with citations……………………….……..…..12

Table S7: Quality Assessment ratings……………….…………………………………………………………………20

Table S1: Electronic database search terms optimised for EMBASE.

| # | Terms |
| --- | --- |
| 1. | exp executive function/ |
| 2. | ("executive function*" or "executive control" or “cognitive control” or “executive dysfunction”).mp. |
| 3. | exp working memory/ |
| 4. | exp problem solving/ or planning.mp. |
| 5. | exp "inhibition (psychology)"/ |
| 6. | ("cognitive flexibility" or "mental flexibility").mp. |
| 7. | ("inhibitory control" or “interference control”).mp. |
| 8. | updating.mp. |
| 9. | "fluid reasoning".mp. |
| 10. | (shift* or switch* or "task-switching" or “set-shifting”).mp. |
| 11. | ("attention control" or “attentional control” or “executive attention”).mp. |
| 12. | exp preschool child/ or (preschool* or pre-school* or “early childhood” or “young child”).mp. |
| 13. | 1 or 2 or 3 or 4 or 5 or 6 or 7 or 8 or 9 or 10 or 11 |
| 14. | exp autism/ or exp Asperger syndrome/ |
| 15. | (autis*or “ASD” or “autism spectrum disorder” or “pervasive developmental disorder” or Asperger*).mp. |
| 16. | ADHD.mp. or exp attention deficit disorder/ |
| 17. | ("Attention deficit hyperactivity disorder" or "Attention deficit disorder with hyperactivity").mp. |
| 18. | 14 or 15 |
| 19. | 16 or 17 |
| 20. | 18 or 19 |
| 21. | 12 and 13 and 20 |
| 22. | limit 21 to (english language and embase and english and (article or article in press) and journal and child <1-6> and children) |

Table S2: Electronic database search terms optimised for MEDLINE and PubMed.

| # | Terms |
| --- | --- |
| 1. | exp Executive Function/ |
| 2. | ("executive function*" or "executive control" or “cognitive control” or “executive dysfunction”).mp. |
| 3. | "working memory".mp. |
| 4. | exp Problem Solving/ or planning.mp. |
| 5. | exp Inhibition, Psychological/ |
| 6. | ("cognitive flexibility" or "mental flexibility").mp. |
| 7. | ("inhibitory control" or “interference control”).mp. |
| 8. | updating.mp. |
| 9. | "fluid reasoning".mp. |
| 10. | (shift* or switch*).mp. |
| 11. | ("attention control" or “attentional control” or “executive attention”).mp. |
| 12. | exp Child, Preschool/ or (preschool* or pre-school* or “early childhood” or “young child*”).mp. |
| 13. | 1 or 2 or 3 or 4 or 5 or 6 or 7 or 8 or 9 or 10 or 11 |
| 14. | exp Autistic Disorder/ or exp Autism Spectrum Disorder/ or exp Asperger Syndrome/ or exp Child Development Disorders, Pervasive/ |
| 15. | (autis*or Asperger*).mp. |
| 16. | exp Attention Deficit Disorder with Hyperactivity/ or adhd.mp. |
| 17. | "Attention Deficit Hyperactivity Disorder".mp. |
| 18. | 14 or 15 |
| 19. | 16 or 17 |
| 20. | 18 or 19 |
| 21. | 12 and 13 and 20 |
| 22. | limit 21 to (english language and "all child (0 to 18 years)" and english and journal article and medline and children) |

.

Table S3: Electronic database search terms optimised for PsycINFO.

| # | Terms |
| --- | --- |
| 1. | exp Executive Function/ |
| 2. | ("executive function*" or "executive control" or “cognitive control” or “executive dysfunction”).mp. |
| 3. | "working memory".mp. |
| 4. | exp Problem Solving/ or ("problem-solving" or planning).mp. |
| 5. | exp Cognitive Control/ |
| 6. | exp Cognitive Flexibility/ or "mental flexibility".mp. |
| 7. | exp Response Inhibition/ or ("inhibitory control" or "interference control").mp. |
| 8. | updating.mp. |
| 9. | "fluid reasoning".mp. |
| 10. | ("set-shifting" or shift*).mp. or exp Set Shifting/ |
| 11. | ("attention* control" or “executive attention”).mp. |
| 12. | exp Task Switching/ or switch*.mp. |
| 13. | exp exp Preschool Students/ or (preschool* or pre-school* or “early childhood” or “young child*”).mp. |
| 14. | 1 or 2 or 3 or 4 or 5 or 6 or 7 or 8 or 9 or 10 or 11 or 12 |
| 15. | exp Autism Spectrum Disorders/ |
| 16. | (autis*or “ASD” or Asperger* or "pervasive developmental disorder").mp. |
| 17. | adhd.mp. or exp Attention Deficit Disorder with Hyperactivity/ |
| 18. | "Attention Deficit Hyperactivity Disorder".mp. |
| 19. | 15 or 16 |
| 20. | 17 or 18 |
| 21. | 19 or 20 |
| 22. | 13 and 14 and 21 |
| 23. | limit 22 to (english language and childhood <birth to 12 years> and 100 childhood <birth to age 12 yrs> and "0110 peer-reviewed journal" and english and human) |

Table S4: Electronic database search optimised for Web of Science.

| Search Terms |
| --- |
| Executive Function* OR Executive Control* OR "Executive dysfunction" OR "Cognitive Control" OR “cognitive flexibility” OR “mental flexibility” OR “Problem solving” OR “problem-solving” OR Inhibition OR “inhibitory control” OR “interference control” OR "Working memory" OR updating OR “fluid reasoning” OR switch* OR planning OR shift* OR “task-switching” OR “set-shifting” OR "attention control" OR “executive attention” OR attention* |
| AND  pre-school* OR preschool* OR “early childhood” OR “young child*” |
| AND  (ADHD OR “Attention Deficit Hyperactivity Disorder” OR “Attention deficit disorder with hyperactivity”) OR (autis* OR "ASD" OR “autism spectrum disorder” OR Asperger* OR “pervasive developmental disorder") |
| NOT  intervention OR training OR treatment OR therapy |
| Limits: Journal article |

Table S5: Recruitment and diagnosis details for included studies.

| Author (year) | Diagnosis criteria and recruitment information | Setting |
| --- | --- | --- |
| ASD studies | | |
| Gardiner et al. (2017) | Prior diagnosis based on DSM-IV-TR, confirmed by ADI-R, ADOS and the ASRS.  IQ >70. | Recruited in Canada. |
| Kimhi et al. (2014) | Prior diagnosis based on DSM-IV-TR, confirmed by the ADI-R.  IQ ≥75 | Recruited through preschool teachers (Israel). |
| Griffith et al. (1999) | Prior diagnosis, verified by ADI-R. | Recruited from an autism clinic (USA). |
| Valeri et al. (2019) | Diagnosis based on DSM-V criteria, the ADI-R and/or ADOS-G.  IQ ≥ 85. | Recruited from a paediatric hospital (Italy). |
| Buzzell et al. (2021) | Previous diagnosis of ASD, confirmed with the ADOS-2.  IQ ≥ 85 | Setting and recruitment process unclear. |
| DeLucia et al. (2021) | Parent-reported ASD diagnoses confirmed by the ADOS-2. | Recruited through community organisations for children with neurodevelopmental disorders. TD children recruited from local preschools, community centers and Head Start programs (USA). |
| McEvoy et al. (1993) | Prior diagnosis based on DSM-III-R, confirmed by the CARS. | Recruited from a child development clinic (USA). |
| Yerys et al. (2007) | Previous diagnosis confirmed by the ADI-R, ADOS-G, and DSM-IV checklist.  MA≥18 months. | Recruited from specialty clinics, parents/advocacy groups and community services (USA). |
| Jahromi et al. (2013) | Clinical diagnosis confirmed with the ADI-R. | Recruited from preschool and autism resource centre (USA). |
| Pellicano et al. (2006) | Prior diagnosis based on DSM-IV, verified by the ADI-R.  IQ ≥80. | Recruited through an autism register, early intervention agencies, speech pathologists and support groups (Australia). |
| Pellicano (2007) | Prior diagnosis based on DSM-IV, verified by the ADI–R.  IQ ≥80. | Recruited from early intervention agencies, support groups, speech therapists, paediatricians |
| Garon et al. (2018) | Prior diagnosis of ASD confirmed by DSM-IV-TR criteria, ADOS and ADI-R. | Recruited through a health centre (Canada). |
| Smithson et al. (2013) | Prior diagnosis based on DSM-IV-TR, detailed history, observation, and the ADOS. | Clinically referred sample (USA). |
| Dawson et al. (2002) | Diagnosis based on DSM-IV, clinical judgment, ADOS-G and ADI-R. | Recruited from schools, community groups, clinics and hospitals (USA). |
| Zhao et al. (2019) | Prior diagnosis based on DSM-IV-TR, the Chinese version of the ASQ and SRS. | Recruited from an inclusive kindergarten (China). |
| Pellicano et al. (2017) | Independent diagnosis based on ICD-10/DSM-IV, the SCQ and ADOS-G.  IQ ≥ 70. | Recruited from community contacts (UK). |
| Smith et al. (2019) | Diagnosis based on the ADOS-2. | Recruited through early intervention agencies (USA). |
| Fanning et al. (2018) | Prior diagnosis confirmed through expert clinical judgment and ADOS-2. | Recruited from an early learning and care centre for autism (Australia). |
| Leekam et al. (2000) | Diagnosis based on DSM-III-R/DSM-IV/ ICD-10. Majority diagnosed through ADI-R. | Recruited from diagnostic and educational services (UK). |
| Rutherford & Rogers (2003) | Prior diagnosis based on the DSM-IV, ADI-R, ADOS-G. | Recruited from health and early education agencies (USA). |
| Stahl & Pry (2002) | Prior diagnosis based on ICD-10, confirmed by the ADI-R. | Recruited in France. |
| ADHD studies | | |
| Çak et al. (2017) | Diagnosis based on DSM-IV, confirmed by K-SADS-PL and CPRS-R/S.  IQ ≥80. Children on special education programmes excluded. | Recruited from outpatient clinic (Turkey). |
| Schneider et al. (2020) | Diagnosis based on DSM-IV-TR, DISC-YC, DICA-IV, CPRS-R/CTRS-R. IQ ≥80. Exclusions: history of abuse, language/ psychiatric disorder (apart from ODD and Adjustment Disorder). | Recruited from community, paediatricians (USA). |
| Lacerda et al. (2020) | Diagnosis based on K-SADS-PL.  IQ ≥50. | Children previously hospitalised at neonatology unit (Brazil). |
| Schneider et al. (2016) | Diagnosis based on DSM-IV, DISC-YC, DICA-IV, CPRS-R.  IQ ≥80. Exclusions: history of abuse, language disorder or any psychiatric disorder apart from ODD and Adjustment Disorder. | Recruited from community, paediatricians (USA). |
| Zhang et al. (2018) | Diagnosis based on DSM‑V, confirmed by the DIPA.  IQ ≥80. | Recruited from an outpatient clinic (China). |
| Mariani et al. (1997) | Diagnosis based on DSM-III-R and the CPRS-R or the CTRS-R.  IQ >80. | Recruited from referrals to an ADHD clinic (USA). |
| Mahone & Hoffman (2007) | Independent diagnosis based on DSM-IV, confirmed by CPRS-R.  Verbal IQ ≥80. | Recruited from preschools, day centres, outpatient clinics (USA). |
| Sjöwall & Thorell (2019) | Diagnosis based on DSM-V, ADHD Rating Scale IV. IQ ≥70. | Clinically referred sample (Sweden). |
| Dalen et al. (2004) | Diagnosis based on the PACS. | Identified by health visitors (UK). |
| Schoemaker et al. (2012) | Diagnosis based on DSM–IV–TR, CBCL, C-TRF, functioning, structured observation.  IQ ≥70. | Recruited from outpatient clinic (The Netherlands). |

*Note.* ADHD Rating Scale IV: DuPaul et al., 1998; ADI-R: Autism Diagnostic Interview–Revised; ADOS: Autism Diagnostic Observation Schedule; ASQ: Autism Spectrum Quotient (Auyeung et al., 2008); ASRS: Autism Spectrum Rating Scales (Goldstein & Naglieri, 2009); CARS: Childhood Autism Rating Scale (Schopler et al., 1986); CPRS-R: Conners’ Parent Rating Scale – Revised and CTRS-R: Conners’ Teacher Rating Scale – Revised (Conners, 1997); DICA-IV: Diagnostic Interview for Children and Adolescents -fourth edition (Reich et al., 1997); DISC-YC: Diagnostic Interview Schedule for Children-Young Child version (Lucas et al., 1998, 2008); DIPA: Diagnostic Infant and Preschool Assessment (Scheeringa & Haslett, 2010); DSM: Diagnostic and Statistical Manual of Mental Disorders; ICD-10: International Classification of Diseases -10^th^ Version (World Health Organization, 1993); IQ: Intelligence Quotient; K-SADS-PL: Schedule for Affective Disorders and Schizophrenia for School-Age Children-Present and Lifetime (Kaufman et al., 1997); PACS: Parental Account of Childhood Symptoms (Taylor et al., 1986); SCQ: Social Communication Questionnaire (Rutter et al., 2003); SRS: Social Responsiveness Scale (Constantino & Gruber, 2002).

**References**

American Psychiatric Association. (2013). Diagnostic and statistical manual of mental health disorders (5th ed.).

Auyeung, B., Baron-Cohen, S., Wheelwright, S., & Allison, C. (2008). The autism spectrum quotient: Children’s version (AQ-Child). *Journal of Autism and Developmental Disorders, 38*(7), 1230– 1240.

Conners, C. K. (1997). *Conners’ rating scales - revised technical manual*. Multi-Health Systems Inc.

Constantino, J. N., & Gruber, C. P. (2002). *The social responsiveness scale*. Western Psychological Services

DuPaul, G. J., Power, T. J., Anastopoulos, A. D., & Reid, R. (1998). *ADHD rating scale- IV. Checklists, norms, and clinical interpretation.* Guilford Press.

Goldstein, S., & Naglieri, J. A. (2009). *ASRS: Autism Spectrum Rating Scales*. Multi-Health Systems.

Kaufman J., Birmaher B., Brent D., Rao U., Flynn C., Moreci P., et al. (1997). Schedule for Affective Disorders and Schizophrenia for School-Age Children-Present and Lifetime Version (K-SADS-PL): initial reliability and validity data. *J Am Acad Child Adolesc Psychiatry, 36*(7), 980-988.

Lucas, C. P., Fisher, P., & Luby, J. L. (1998). *Young child DISC-IV research draft: Diagnostic interview schedule for children* (Division of Children Psychiatry, Joy and William Ruane Center to Identify and Treat Mood Disorders). Columbia University.

Lucas, C. P., Fisher, P., & Luby, J. L. (2008). *Young child DISC-IV: Diagnostic interview schedule for children.* Columbia University, Division of Children Psychiatry, Joy and William Ruane Center to Identify and Treat Mood Disorders.

Reich, W., Welner, Z., & Herjanic, B. (1997). *The diagnostic interview for children and adolescents- IV*. Multi-Health Systems.

Rutter, M., Bailey, A., & Lord, C. (2003). *The social communication questionnaire: Manual*. Western Psychological Services.

Scheeringa M. S., Haslett N. (2010). The reliability and criterion validity of the diagnostic infant and preschool assessment: A new diagnostic instrument for young children. *Child Psychiatry Hum Dev, 41*(3), 299‑312.

Schopler, E., Reichler, R. J., & Renner, B. R., (1986). *The Childhood Autism Rating Scale* (CARS). Irvington.

Taylor, E., Everitt, B., Thorley, G., Schachar, R., Rutter, M., & Wieselberg, M. (1986). Conduct disorder and hyperactivity: II. A cluster analytic approach to the identification of a behavioural syndrome. *British Journal of Psychiatry, 149*(6), 768-77.

World Health Organization, (1993). The ICD-10 Classification of Mental and Behavioural Disorders. Available at: www.who.int/entity/classifications/icd/en/bluebook.pdf; 1: 1-263. Accessed March 2020.

Table S6: Laboratory-based tasks in included studies with citations

| Task | Included in: | Psychometric properties previously assessed? | Used with ASD/ADHD? |
| --- | --- | --- | --- |
| Flexible Item Selection Task (Jacques & Zelazo, 2001) | Kimhi et al. (2014) | Validated in preschoolers, good validity but unclear reliability | Used with school-aged children with ASD (e.g. Yerys et al., 2012) |
| Tower of London (Shallice, 1982) | Kimhi et al. (2014), Pellicano et al. (2006), Pellicano (2007): | Good validity and when administered in older children but low reliability (Bishop et al., 2002) | Used with older children with ASD (e.g. Robinson et al., 2009) |
| Boxes task, A-not-B, A-not-B with Invisible Displacement (Diamond et al., 1997),  Object Retrieval from transparent boxes (Diamond, 1991),  Spatial Reversal (Kaufman et al., 1989) | Griffith et al. (1999), Yerys et al. (2007), Rutherford & Rogers (2003), Dawson et al. (2002), McEvoy et al. (1993) | Validity previously assessed.  Reliability of tasks unclear. McEvoy et al. (1993) video-recorded and double-scored tasks and reported good inter-rater reliability. | A-not-B and Boxes tasks originally developed and mainly used for infants and non-human primates. Not commonly used with children with ASD prior to included studies. |
| Multi-step multi-location task based on Zelazo et al. (1998) | Stahl & Pry (2002) | Task simplified and no data was provided on validity/reliability. | Original task developed for typically developing two-year old children. |
| Delayed Non-Matching to Sample (Diamond et al., 1999), Object Discrimination Reversal (Butter, 1969) | Dawson et al. (2002) | Reliability and validity of tasks unclear. | Tasks used with non-human primates and infants. |
| Luria’s hand-game (Hughes, 1996),  Mazes task (WPPSI-R; Wechsler, 1989),  Teddy bear card-sorting task (Hughes, 1998) | Pellicano et al. (2006) and Pellicano (2007) | Tasks previously validated but unclear test-retest reliability. Good validity and reliability reported for the Mazes task. | Luria’s hand-game previously used with children with autism.  Other tasks used with typically developing preschool children. |
| Stroop Night and Day task, Pattern Making test, Card Sort, Spin the Pots.  Tasks from BAFE: Italian neuro-psychological battery for preschoolers (Valeri et al., 2015) | Valeri et al. (2019) | Tasks previously validated in preschoolers and reported to have good reliability and validity. | Previously used with typically developing preschoolers. |
| Night/Day inhibition task (Gerstadt et al., 1994) | Jahromi et al. (2013)  DeLucia et al. (2019) | Task previously validated for use in preschool children. | Previously used with children with autism (e.g. Russell et al., 1999) |
| Corsi Blocks (Corsi, 1973) | Pellicano et al. (2017) | Previously validated task for older children and acceptable reliability reported for children with autism. | Previously used with older children with autism (Morsanyi & Holyoak, 2010). |
| Windows Task (Rogers & Wehner, 1997) | Yerys et al. (2007) | Tasks was simplified for this study and data on reliability and validity have not been reported. | Original task used previously with preschool children with autism (Russell et al., 1991) |
| Delayed Response task (Diamond & Goldman-Rakic, 1985) | McEvoy et al. (1993) | Task validated for use in non-human primates. Inter-rater reliability assessed and reported to be good in this study. | Used with non-human primates and infants. |
| Hide and seek, Tricky box, Flap book. Tasks from Preschool executive function battery (Garon et al., 2014). | Garon et al. (2018) | Internal reliability was assessed in study, validity was assessed previously in preschoolers. | Used with typically developing preschool children. |
| Monkey Tower, Boy-Girl Stroop, Go/No-Go task (Kerns & McInerney, 2007)  Preschool Continuous Performance Test (Kerns & Rondeau, 1998), | Gardiner et al. (2017) | Previously validated tasks for use in preschool and older children and demonstrated to have good reliability and validity. | Used with typically developing preschool children. |
| Dimensional Change Card Sorting task (Zelazo, 2006). | Zhao et al. (2019), Pellicano et al. (2017) | Previously validated task for use in children (preschool and older). | Previously used with children with ASD (e.g. Yi et al., 2012) |
| Continuous Performance Task-Preschool (Mahone et al., 2001)  Auditory Working Memory (Woodcock Johnson III; Woodcock et al., 2001)  Spatial Working Memory and Stop Signal Response Time Task (CANTAB; CeNeS Cognition, 1996)  Conflicting Motor Response Test (Luria- Christensen Battery; Christensen, 1975) | Schneider et al. (2016) | Tasks have been previously validated for use with children. | All tasks have been previously used with children with ADHD |
| Statue (NEPSY-II; Korkman et al., 2007) | Zhang et al. (2018), Schneider et al. (2016) | Task previously validated for use with children. | Task previously used with young children with ADHD (e.g. Breaux et al., 2016) |
| Go/No-Go Inhibition ‘Puppet says…’ task (Kochanska et al. 1996)  Set shifting-modified Weigl block sorting task (De Renzi et al., 1966) | Dalen et al. (2004) | Tasks previously validated (Weigl task initially developed for lesion patients). Satisfactory/good test-retest reliability reported for both. | Go/No-go task used with preschool children, Weigl block sorting task used with preschool children with ADHD symptoms (e.g. Sonuga-Barke et a., 2003) |
| K-ABC Number Recall Test and K-ABC Spatial Memory Test (Kaufman & Kaufman, 1983),  Porteus Mazes (Porteus, 1965)  Colour Form Test (Reitan & Wolfson, 1985),  Continuous Performance Test (Gordon, 1983). | Mariani et al. (1997) | Tasks previously validated for use in children. Order of test administration not identical across participants and might have introduced order effects, behavioural coder not blind to group membership. | All tasks apart from Colour Form Test have been previously used with children with ADHD. |
| Go/No-Go, Shape School – Inhibit Condition, Nine Boxes and Delayed Alternation. Tasks adapted from Wiebe et al., (2011). | Schoemaker et al. (2012) | Tasks previously validated for use with preschool children, however low reliability was reported for Delayed Alternation and Nine Boxes in previous studies. | Tasks previously used with children with ADHD symptoms. |
| Backward Digit Span (WISC-IV; Wechsler, 2003),  Find the Phone task (Sjöwall et al. 2013) | Sjöwall & Thorell (2019) | Tasks previously validated for use with children, tasks were double-coded and inter-rater reliability was assessed in study. | Tasks previously used with older children with ADHD. |
| Go/No-Go Zoo game (McDermott et al., 2014; Lamm et al., 2014) | Buzzell et al. (2021) | Task validated previously in young children | No, only used with typically developing children |
| The Conners’ Kiddie Continuous Performance Test (Conners, 2001) | Çak et al. (2017), Lacerda et al. (2020) | Task validated in preschoolers | Used previously in studies of preschoolers with ADHD symptoms (e.g. Berwid et al., 2005) |

**References**

Berwid, O. G., Curko Kera, E. A., Marks, D. J., Santra, A., Bender, H. A., & Halperin, J. M. (2005). Sustained attention and response inhibition in young children at risk for attention deficit/hyperactivity disorder*. Journal of Child Psychology and Psychiatry, 46,* 1219-1229.

Bishop, D. V. M., Aamodt-Leeper, G., Creswell, C., McGurk, R., & Skuse, D. (2002). Individual differences in cognitive planning on the Tower of Hanoi task: Neuropsychological maturity or measurement error? *Journal of Child Psychology and Psychiatry*, *42*, 551–556.

Breaux, R. P., Griffith, S. F., & Harvey, E. A. (2016). Preschool neuropsychological measures as predictors of later attention deficit hyperactivity disorder. *Journal of abnormal child psychology*, *44*(8), 1455-1471.

Butter, C. M. (1969). Perseveration in extinction and in discrimination reversal tasks following selective frontal ablations in Macaca mulatta*. Physiology and Behavior, 4*(2), 163– 171.

Christensen, A. (1975). *Luria’s neuropsychological investigation.* Spectrum

CeNes Cognition (1996). *CANTAB*. CeNeS Limited.

Conners C. K. (2001*). Conners’ Kiddie Continuous Performance Test (K-CPT).* Multi-Health Systems.

Corsi, P. M. (1973). *Human memory and the medial temporal region of the brain*. ProQuest Information & Learning.

De Renzi E, Faglioni P, & Savoiardo M. (1966). The influence of aphasia and of the hemispheric side of the cerebral lesion on abstract thinking. *Cortex, 2*(4), 399-420.

Diamond, A, & Goldman-Rakic, P, S, (1985). Evidence for involvement of prefrontal cortex in cognitive changes during the first year of life: comparison of human infants and rhesus monkeys on a detour task with transparent barrier. *Society for Neurosciences Abstracts (Part II), 11*, 832.

Diamond, A. (1991). Frontal lobe involvement in cognitive changes during the first year of life. In K. R. Gibson and A. C. Petersen (Eds.), *Brain maturation and cognitive development* (pp. 127–180). Aldine DeGruyter.

Diamond, A., Churchland, A., Cruess, L., & Kirkham, N. Z. (1999). Early developments in the ability to understand the relation between stimulus and reward*. Developmental Psychology, 35*(6), 1507–1517.

Diamond, A., Prevor, M. B., Callender, G., & Druin, D. P. (1997). Prefrontal cortex cognitive deficits in children treated early and continuously for PKU. *Monographs of the Society for Research in Child Development,* i-206.

Garon, N., Smith, I.M., & Bryson, S.E. (2014). A novel executive function battery for preschoolers: Sensitivity to age differences. *Child Neuropsychology, 20*(6), 713–736. doi:10.1080/09297049.2013.857650

Gerstadt, C., Hong, Y., & Diamond, A. (1994). The relationship between cognition and action: Performance of children 3 ½–7 years old on a Stroop-like day-night test. *Cognition, 53*(2), 129–153.

Gordon, M. (1983). *The Gordon Diagnostic System*. Gordon Systems.

Hughes, C. (1996). Control of action and thought: Normal development and dysfunction in autism: A research note. *Journal of Child Psychology and Psychiatry, 37*(2), 229–236.

Hughes, C. (1998). Executive function in preschoolers: Links with theory of mind and verbal ability. *British Journal of Developmental Psychology, 16*(2), 233–253.

Jacques, S., & Zelazo, P. D. (2001). The flexible item selection task (FIST): A measure of executive function in preschoolers. *Developmental Neuropsychology, 20*(3), 573–591.

Kaufman, A. S., & Kaufman, N. L. (1983). *Kaufman Assessment Battery for Children—Interpretive manual.* American Guidance Service

Kaufman, P., Leckman, J., & Ort, S. (1989). Delayed response performance in males with Fragile X. *Journal of Clinical and Experimental Neuropsychology, 12,* 69.

Kerns, K. A., & McInerney, R. (2007). Preschool tasks [Computer software]. University of Victoria.

Kerns, K. A., & Rondeau, L. A. (1998). Development of a continuous performance test for preschool children. *Journal of Attention Disorders, 2*(4), 229–238. doi:10.1177/108705479800200403

Kochanska G., Murray K., Jacques T. Y., Koenig A. L., & Vandegeest A. (1996). Inhibitory control in young children and its role in emerging internalization. *Child Development, 67*(2), 490-507.

Korkman, M., Kirk, U., & Kemp, S. (2007). NEPSY (2nd ed.). Pearson.

Lamm C., Walker O.L., Degnan K.A., Henderson H.A., Pine D.S., McDermott J.M., Fox N.A. (2014). Cognitive control moderates early childhood temperament in predicting social behavior in 7-year-old children: an ERP study. *Dev Sci, 17*, 667-681.

Mahone, E. M., Pillion, J. P., & Hiemenz, J. R. (2001). Initial development of an auditory continuous performance test for preschoolers*. Journal of Attention Disorders, 5*(2), 93–106. doi:10.1177/108705470100500203

McDermott, J.M., Henderson, H.A., Degnan, K.A., Fox, N.A. (2014). Behavioral inhibition and inhibitory control: Independent and interactive effects on socio-emotional behavior in young children (in preparation).

Morsanyi, K., & Holyoak, K. J. (2010). Analogical reasoning ability in autistic and typically developing children. *Developmental science*, *13*(4), 578-587.

Porteus, S. D. (1965). *Porteus Maze Test: Fifty years application*. Psychological Corporation.

Reitan, R. M., & Wolfson, D. (1985). *The Halstead-Reitan Neuropsychological Test Battery*. Neuropsychological Press.Rogers, S. J., & Wehner, E. A. (1997). Modification of the Windows task for young children. Unpublished data.

Robinson, S., Goddard, L., Dritschel, B., Wisley, M., & Howlin, P. (2009). Executive functions in children with autism spectrum disorders. *Brain and cognition, 71(3),* 362-368.

Russell, J., Jarrold, C., & Hood, B. (1999). Two intact executive capacities in children with autism: Implications for the core executive dysfunctions in the disorder. *Journal of Autism and Developmental Disorders*, *29*(2), 103-112.

Russell, J., Mauthner, N., Sharpe, S., & Tidswell, T. (1991). The ‘windows task’as a measure of strategic deception in preschoolers and autistic subjects. *British journal of developmental psychology*, *9*(2), 331-349.

Shallice, T. (1982). Specific impairments of planning. *Philosophical Transactions of the Royal Society of London, 298*(1089), 199–209.

SjöWall, D., Roth, L., Lindqvist, S., & Thorell, L. B. (2013). Multiple deficits in ADHD: Executive dysfunction, delay aversion, reaction time variability and emotional deficits. *Journal of Child Psychology and Psychiatry, 54*(6), 619–627.

Sonuga-Barke, E. J., Dalen, L., & Remington, B. (2003). Do executive deficits and delay aversion make independent contributions to preschool attention-deficit/hyperactivity disorder symptoms? *Journal of the American Academy of Child & Adolescent Psychiatry*, *42*(11), 1335-1342.

Valeri, G., Stievano, P., Ferretti, M. L., Mariani, E., & Pieretti, E. (2015). *BAFE Batteria per l’Assessment delle Funzioni Esecutive.* Hogrefe Editore.

Wechsler, D. (1989). *Wechsler Preschool and Primary Scale of Intelligence—Revised.* Psychological Corporation.

Wechsler, D. (2003*). Wechsler intelligence scale for children-fourth edition*. The Psychological Corporation.

Wiebe, S.A., Sheffield, T., Nelson, J.M., Clark, C.A.C., Chevalier, N., & Espy, K.A. (2011). The structure of executive function in 3-year-olds. *Journal of Experimental Child Psychology, 108*(3), 436–452.

Woodcock, R., McGrew, K., & Mather, N. (2001). *Woodcock-Johnson test of cognitive abilities* (3rd ed.) (WJ III). Riverside Publishing.

Yerys, B. E., Wolff, B. C., Moody, E., Pennington, B. F., & Hepburn, S. L. (2012). Brief report: Impaired Flexible Item Selection Task (FIST) in school-age children with autism spectrum disorders. *Journal of autism and developmental disorders*, *42*(9), 2013-2020.

Yi, L., Liu, Y., Li, Y., Fan, Y., Huang, D., & Gao, D. (2012). Visual scanning patterns during the dimensional change card sorting task in children with autism spectrum disorder. *Autism Research and Treatment*, *2012*.

Zelazo, P. D. (2006). The Dimensional Change Card Sort (DCCS): A method of assessing executive function in children. *Nature Protocols, 1*(1), 297–301.

Zelazo, P. D., Reznick, J. S., & Spinazzola, J. (1998). Representational flexibility and response control in a multistep multilocation search task. *Developmental Psychology, 34*(2), 203.

Table S7: Quality assessment ratings.

| ASD/ ADHD | Author | Were the criteria for inclusion in the sample clearly defined? | Were the study subjects and the setting described in detail? | Were objective, standard criteria used for measuring the condition? | Were confounding factors identified? | Were strategies to deal with confounding factors stated? | Were the outcomes measured in a valid and reliable way? | Was appropriate statistical analysis used? | Score /7 |
| --- | --- | --- | --- | --- | --- | --- | --- | --- | --- |
| ADHD | Çak et al. (2017) | Yes | Yes | Yes | Yes | Yes | Yes | Yes | 7/7 |
| ADHD | Schneider et al. (2020) | Yes | Yes | Yes | Yes | Yes | Yes | Yes | 7/7 |
| ADHD | Lacerda et al. (2020) | Yes | Yes | Yes | Yes | Yes | Unclear | Yes | 6/7 |
| ADHD | Mariani et al. (1997) | Yes | Yes | Yes | Yes | Yes | Unclear | Yes | 6/7 |
| ASD | Gardiner et al. (2017) | Yes | Unclear | Yes | Yes | Yes | Yes | Yes | 6/7 |
| ADHD | Zhang et al. (2018) | Yes | Yes | Yes | Yes | Unclear | Yes | Yes | 6/7 |
| ADHD | Schneider et al. (2016) | Yes | Yes | Yes | Yes | Unclear | Yes | Yes | 6/7 |
| ASD | Kimhi et al. (2014) | Unclear | Yes | Yes | Yes | Yes | Unclear | Yes | 5/7 |
| ASD | Griffith et al (1999) | Unclear | Yes | Yes | Yes | Yes | Unclear | Yes | 5/7 |
| ASD | Valeri et al. (2019) | Yes | Yes | Yes | Unclear | Unclear | Yes | Yes | 5/7 |
| ASD | Buzzell et al. (2021) | Yes | Unclear | Yes | Yes | Yes | Unclear | Yes | 5/7 |
| ASD | DeLucia et al. (2021) | Unclear | Yes | Yes | Yes | Yes | Unclear | Yes | 5/7 |
| ASD | McEvoy et al (1993) | Unclear | Yes | Yes | Yes | Yes | Yes | No | 5/7 |
| ASD | Yerys et al. (2007) | Yes | Yes | Yes | Yes | No | Unclear | Yes | 5/7 |
| ASD | Jahromi et al. (2013) | Unclear | Yes | Yes | Unclear | Unclear | Yes | Yes | 4/7 |
| ASD | Pellicano (2007) | Yes | Yes | Yes | Unclear | Unclear | Unclear | Yes | 4/7 |
| ASD | Pellicano et al. (2006) | Yes | Yes | Yes | Unclear | Unclear | Unclear | Yes | 4/7 |
| ASD | Garon et al. (2018) | Unclear | Yes | Yes | Unclear | Unclear | Yes | Yes | 4/7 |
| ASD | Smithson et al. (2013) | Unclear | Yes | Yes | Unclear | Unclear | Yes | Yes | 4/7 |
| ASD | Dawson et al. (2002) | Yes | Yes | Yes | Yes | Unclear | Unclear | No | 4/7 |
| ASD | Zhao et al. (2019) | Unclear | Yes | Yes | Unclear | No | Yes | Yes | 4/7 |
| ASD | Pellicano et al., (2017) | Unclear | Yes | Yes | Unclear | No | Yes | Yes | 4/7 |
| ASD | Smith et al. (2019) | Unclear | Yes | Yes | Unclear | Unclear | Unclear | Yes | 4/7 |
| ADHD | Mahone & Hoffman (2007) | Yes | No | Yes | Unclear | Unclear | Yes | Yes | 4/7 |
| ADHD | Sjöwall & Thorell (2019) | Unclear | Unclear | Yes | Unclear | Unclear | Yes | Yes | 3/7 |
| ADHD | Dalen et al. (2004) | Unclear | No | Yes | Unclear | Unclear | Yes | Yes | 3/7 |
| ASD | Fanning et al. (2018) | Yes | Yes | No | Unclear | No | No | Yes | 3/7 |
| ADHD | Schoemaker et al. (2012) | Unclear | Yes | Yes | Unclear | Unclear | Unclear | Unclear | 2/7 |
| ASD | Leekam et al. (2000) | Unclear | No | Yes | Unclear | Unclear | Unclear | Yes | 2/7 |
| ASD | Rutherford & Rogers (2003) | Unclear | No | Yes | No | No | Unclear | Yes | 2/7 |
| ASD | Stahl & Pry (2002) | No | No | Yes | No | No | Unclear | Yes | 2/7 |

*Note*. Only items with a Yes rating counted towards the total score.

**Quality Assessment and Risk of Bias**

The Joanna Briggs Institute (JBI) - Critical Appraisal Checklist for Analytical Cross Sectional Studies (Joanna Briggs Institute, 2016; Moola et al., 2017) was approved after extensive peer review by the JBI Scientific Committee. It contains eight items assessing: 1) study eligibility criteria (clearly stated for all groups), 2) description of sample and setting (recruitment process, sample demographics), 3) measurement of exposure, 4) measurement of participant condition (standardised diagnostic methods/criteria used consistently), 5) identification of confounding factors, 6) control of confounding factors, 7) assessment of outcomes (both in terms of validity and reliability; if the task was novel or modified and not previously validated, an assessment of its psychometric properties should be reported), and 8) suitability of statistical analysis (appropriateness of the analytical strategy also in relation to the assumptions associated with the approach, correction for multiple comparisons where appropriate) . Each item is rated as Yes, No, Unclear, or Not Applicable. For the purposes of this review, item 3 relating to the measurement of the exposure was dropped as it was deemed non-applicable. Items were rated as Unclear when there was insufficient information in the report to assign a Yes or No, and to indicate a middle rating for studies that partly satisfied the quality criteria. Therefore, a traffic light system (green – Yes, amber – Unclear, red – No) was used to visually present the results of the quality assessment. Only “Yes” ratings counted towards a total quality score.

With regards to potential confounding factors, socioeconomic status (SES), IQ and gender were identified by previous studies to be associated with ASD and ADHD (e.g. Loomes et al., 2017; Scahill, & Schwab-Stone, 2000) and with executive functioning outcomes (though gender less so: Grissom et al., 2019). SES in particular was consistently found to be associated with ASD and ADHD (e.g. Rai et al., 2012; Russell et al., 2016) and with poorer EF outcomes (Sarsour et al., 2011; Hackman et al., 2015), irrespective of IQ (Arán-Filippetti & Richaud de Minzi, 2012). IQ has also been found to be associated with EF (e.g. Mahone at al., 2002), though there is a debate around whether it should be statistically partialled out (see Dennis et al., 2009). The risk of confounding was also related to study specific measures and characteristics the participant groups were unequal on. If studies identified and controlled for some potential confounders but not all, this was given an “Unclear” rating.

The item corresponding to the control of confounders (Item 5) yielded the lowest agreement between raters (kappa=0.48 and 68% agreement). Most of the disagreements were between ‘No’ and ‘Unclear’ ratings when confounders were not clearly controlled for in the analysis or matched between groups. The disagreements were discussed and a consensus rating was agreed for each individual study. Although these disagreements played a role in the Kappa calculation, they did not affect the total quality score, as both ‘No’ and ‘Unclear’ were assigned 0 points. Item 7 also yielded a kappa of 0.48, but there was high agreement on this item (93% - raters only disagreed on two occasions). All other kappa values were higher than 0.70 and agreement rates higher than 83%.

**References**

Aran-Filippetti, V., & Richaud de Minzi, M. C. (2012). A structural analysis of executive functions and socioeconomic status in school-age children: Cognitive factors as effect mediators. *The Journal of genetic psychology, 173*(4), 393-416.

Dennis, M., Francis, D. J., Cirino, P. T., Schachar, R., Barnes, M. A., & Fletcher, J. M. (2009). Why IQ is not a covariate in cognitive studies of neurodevelopmental disorders*. Journal of the International Neuropsychological Society: JINS, 15*(3), 331.

Grissom, N. M., & Reyes, T. M. (2019). Let’s call the whole thing off: evaluating gender and sex differences in executive function. *Neuropsychopharmacology, 44*(1), 86-96.

Hackman, D. A., Gallop, R., Evans, G. W., & Farah, M. J. (2015). Socioeconomic status and executive function: Developmental trajectories and mediation. *Developmental science, 18(5),* 686-702.

Loomes, R., Hull, L., & Mandy, W. P. L. (2017). What is the male-to-female ratio in autism spectrum disorder? A systematic review and meta-analysis. *Journal of the American Academy of Child & Adolescent Psychiatry, 56*(6), 466-474.

Mahone, E. M., Hagelthorn, K. M., Cutting, L. E., Schuerholz, L. J., Pelletier, S. F., Rawlins, C., Singer, H. S., & Denckla, M. B. (2002). Effects of IQ on executive function measures in children with ADHD. *Child Neuropsychology, 8*(1), 52-65.

Moola, S., Munn, Z., Tufanaru, C., Aromataris, E., Sears, K., Sfetcu, R., Currie, M., Qureshi R., Mattis, P., Lisy, K., Mu, P-F. (2017). Chapter 7: Systematic reviews of etiology and risk. In: E. Aromataris, Z. Munn (Eds). *Joanna Briggs Institute Reviewer's Manual.* The Joanna Briggs Institute. Available from https://reviewersmanual.joannabriggs.org/

Rai, D., Lewis, G., Lundberg, M., Araya, R., Svensson, A., Dalman, C., Carpenter, P., & Magnusson, C. (2012). Parental socioeconomic status and risk of offspring autism spectrum disorders in a Swedish population-based study. *Journal of the American Academy of Child & Adolescent Psychiatry, 51*(5), 467-476.

Russell, A. E., Ford, T., Williams, R., & Russell, G. (2016). The association between socioeconomic disadvantage and attention deficit/hyperactivity disorder (ADHD): a systematic review. *Child Psychiatry & Human Development, 47*(3), 440-458.

Sarsour, K., Sheridan, M., Jutte, D., Nuru-Jeter, A., Hinshaw, S., & Boyce, W. T. (2011). Family socioeconomic status and child executive functions: The roles of language, home environment, and single parenthood. *Journal of the International Neuropsychological Society: JINS, 17*(1), 120.

Scahill, L., & Schwab-Stone, M. (2000). Epidemiology of ADHD in school-age children. *Child and adolescent psychiatric clinics of North America, 9*(3), 541-555.
